# Importance of family history and health checkup for school-aged children for type IV collagen-associated nephropathy in hereditary kidney disease

**DOI:** 10.1007/s40620-025-02355-w

**Published:** 2025-08-02

**Authors:** Ryosuke Kawamoto, Takayasu Mori, Motoko Chiga, Takuya Fujimaru, Azuma Nanamatsu, Tamami Fujiki, Yutaro Mori, Shintaro Mandai, Fumiaki Ando, Koichiro Susa, Tatemitsu Rai, Eisei Sohara, Shinichi Uchida

**Affiliations:** Department of Nephrology, Graduate School of Medical and Dental Sciences, Institute of Science Tokyo, 1-5-45 Yushima, Bunkyo, Tokyo 113-8519 Japan

**Keywords:** *COL4A3*, *COL4A4*, *COL4A5*, Alport syndrome, Thin basement membrane disease, School urinalysis

## Abstract

**Background:**

Advancements in genetic analysis have revealed a higher prevalence of hereditary kidney disease than expected. This study focuses on the enrollment and analysis of patients with chronic kidney disease (CKD) and a family history of CKD to identify disease-causing variants. Additionally, by incorporating data from childhood urine tests, the study examines the utility of these screenings in early disease detection.

**Methods:**

An observational study utilizing genetic data and clinical assessments from patients with familial CKD. A total of 85 patients with familial CKD, diagnosed by clinicians and confirmed through genetic testing from 2014 to 2020, were included. Patients with cystic kidney diseases were excluded. The presence of *COL4As* (*COL4A3*, *COL4A4*, *COL4A5*) gene variants and other genetic variants associated with kidney disease was examined using a comprehensive gene panel.

**Results:**

Of the patients, 41.2% had disease-causing variants in *COL4As* variants. The median age at manifestation onset was significantly lower in the *COL4As* group compared to patients with other disease-causing variants or those with no identified disease-causing variants. Early manifestations of microscopic hematuria were notably prevalent, indicating potential early markers for genetic kidney diseases.

**Conclusions:**

The findings underscore the importance of family history in diagnosing genetic kidney diseases and suggest that early genetic testing, coupled with regular monitoring of urinary abnormalities, could significantly improve disease management and outcomes. Further research is necessary to explore comprehensive genetic screening and its integration into routine clinical practice.

**Graphical abstract:**

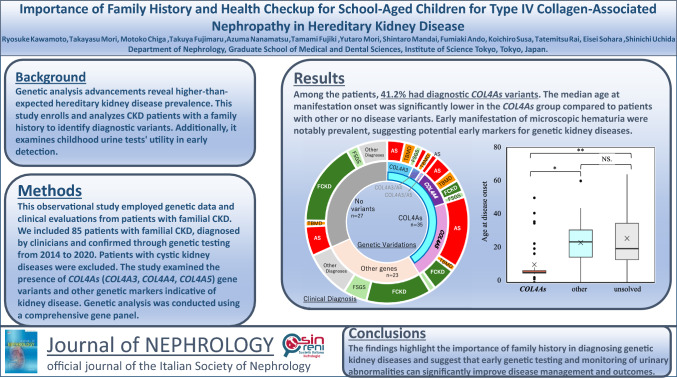

**Supplementary Information:**

The online version contains supplementary material available at 10.1007/s40620-025-02355-w.

## Introduction

Kidney failure, necessitating dialysis or transplantation, significantly impacts patients' quality of life [1] and life expectancy while escalating healthcare costs. As the global population ages, the previously overlooked gradual decline in kidney function is emerging as a significant health concern [2]. Recent breakthroughs in gene analysis have shed light on the etiology of chronic kidney disease (CKD) [3, 4]. Studies such as those by Groopman et al. [5] and Domingo-Gallego et al. [6] have identified major genetic variants in a significant proportion of patients with early-onset CKD, emphasizing the role of specific genes like *PKD1*, *PKD2*, and *COL4As* in disease development. Recent studies, such as that by Dahl et al., demonstrate the significant impact of genetic testing in refining diagnoses and guiding management in CKD, showing that nearly half of the patients had changes in their treatment plans based on genetic insights [7].

Thin basement membrane disease (TBMD), which was historically known as benign familial hematuria [8, 9] and considered to have little clinical significance [10], is now recognized for its potential to progress to CKD [11, 12]. This underscores the variability in clinical outcomes, even among individuals with similar genetic variants [13]. This variability highlights the need for cautious diagnosis, particularly in cases with heterozygous variants where autosomal dominant patterns may be suspected.

This study aimed to explore the prevalence of *COL4As*-related kidney diseases in Japanese patients with a familial history of CKD, utilizing a comprehensive genetic panel (SPEEDI-KID [14]). Additionally, leveraging Japan’s unique health checkup system for school-aged children, we examined the significance of early urine abnormality detection, potentially crucial for early intervention and better management of CKD.

## Methods

### Patients studied

A total of 85 patients clinically diagnosed with familial CKD by physicians who provided clinical information and obtained blood specimens for DNA testing between 2014 and 2020 were enrolled in the study. After anonymization, clinical data such as age at onset, age at examination, sex, clinical diagnosis, microscopic hematuria, urinary protein, renal biopsy results, and family history were collected. The patients were those with a family history of CKD, that is, kidney dysfunction with estimated glomerular filtration (eGFR) < 60 mL/min/1.73 m^2^ (estimates were only adopted for individuals aged 18 and over), sustained proteinuria, or hematuria within the third degree of kinship including the proband. Cystic kidney diseases such as autosomal dominant polycystic kidney disease that can be screened by imaging diagnosis were excluded.

Japan has an obligatory health checkup system for all school-aged children; thus, if abnormal urine examinations such as hematuria or proteinuria were identified while they were still of  school age, the disease onset could be traced relatively accurately. Taking advantage of this feature, disease onset was first confirmed using medical histories in which some clinical abnormalities in urine examination or kidney dysfunction were indicated, and the relationship with disease-causing genes was investigated.

All participants provided written informed consent and agreed that their clinical information and DNA could be used in studies to identify genetic risk variants for kidney function. The Institutional Review Board of the Institute of Science Tokyo approved this study (Approval no. G2000-080).

### Gene panel sequencing

Gene panel sequencing was performed as described previously[14, 15] and in Item S1, Table S1.

### Variant filtering and classification

We reported the Variant Filtering and Classification method in Item S2. “Disease-causing variants,” which are considered potentially pathogenic according to the criteria that corresponds to any of the following, were identified: (a) pathogenic, likely pathogenic variants in 2015 ACMG criteria [16] using InterVar; (b) known disease-causing variants that have been published previously other than (a); and (c) rare variants in responsible genes with “CADD > 20 or null” and “MAF in ToMMo 8.3KJPN (Japanese MAF database) < 0.0005”, other than (a) and (b). Variants that did not fit any of (a–c) were considered benign or of unknown significance.

### Statistical analysis

The Kruskal–Wallis test was used to compare the median values among the groups. Subsequently, post-hoc pairwise comparisons were conducted using Mann–Whitney *U* tests with Bonferroni correction to compare the mean values between specific pairs of groups. The significance level was set at 0.05.

## Results

### Baseline patient characteristics and clinical diagnoses

The characteristics of the patients are shown in Table 1. A detailed family history of each patient is summarized in Table S2. At the time of study entry, the median age was 39 (interquartile range 27–48) years; 42 (49.4%) were female; 51 (60.0%) had eGFR < 60 mL/min/1.73 m^2^; 71 (83.5%) had proteinuria; and 57 (67.1%) had microscopic hematuria. In addition, 32 (37.6%) were clinically pre-diagnosed with Alport syndrome/thin basement membrane disease before genetic testing. Moreover, 30 (35.3%) were diagnosed with familial clustering of kidney disease [17], and 9 (10.6%) were diagnosed with focal segmental glomerulosclerosis (FSGS) based on kidney biopsy, although the underlying disease was unknown prior to genetic analysis. Kidney biopsies were performed in 60 (70.6%) patients, noting Alport syndrome/thin basement membrane disease in 22 (25.9%) and FSGS in 17 (20.0%), and 4 (4.7%) were diagnosed with Alport syndrome/thin basement membrane disease while presenting with FSGS.

**Table 1:** Clinical characteristics of all patients

	Number of patients (percentage)
Total	85
Age at time of study entry	
0–10 years	1 (1.1%)
11–20 years	8 (9.4%)
21–40 years	38 (11.8%)
41–60 years	32 (37.4%)
≥ 61 years	6 (7.1%)
Age at onset	
0–10 years	36 (42.4%)
11–20 years	19 (22.4%)
21–40 years	21 (24.7%)
41–60 years	8 (9.4%)
≥ 61 years	1 (1.1%)
Sex	
Female	42 (49.4%)
Male	43 (50.6%)
eGFR*	
≥ 60 mL/min/1.73 m^2^	34 (40.0%)
< 60 mL/min/1.73 m^2^	51 (60.0%)
Proteinuria*	
Positive	71 (83.5%)
Negative	14 (16.5%)
Microscopic hematuria^a^	
Positive	57 (67.1%)
Negative	28 (32.9%)
Clinical diagnosis	
AS	25 (29.4%)
TBMD	7 (8.2%)
FCKD	30 (35.3%)
FSGS	9 (10.6%)
Others	14 (16.5%)
Biopsy	
Total	60 (70.6%)
AS/TBMD^b^	22 (25.9%)
FSGS^b^	17 (20.0%)
Nephrosclerosis	4 (4.8%)
IgAN^b^	2 (2.4%)
Nephronophthisis	2 (2.4%)
Other^c^	10 (14.1%)
MGA/np	8 (9.4%)
Genes^d^	
* COL4As*	35 [42] (41.2%)
* COL4A3*	9 [12] (10.6%)
* COL4A4*	10 [11] (11.8%)
* COL4A5*	19 [19] (22.4%)
Other than *COL4As*	23 [32] (27.1%)
Undetermined	27 (31.8%)

*AS* Alport syndrome, *TBMD* thin basement membrane disease, *FCKD* familial clustering of kidney disease, *FSGS* focal segmental glomerulonephritis, *IgAN* IgA nephropathy, *MGA/np* minor or no significant glomerular abnormalities

^a^eGFR, urinary protein, and microscopic hematuria were measured at the time of study entry

^b^Four patients had both AS/TBMD and FSGS, and one patient had both IgAN and AS

^c^The "Other" category comprised one case each of membranous nephropathy (MN), Dent disease, type I membranoproliferative glomerulonephritis (MPGN I), chronic interstitial nephritis (CIN), MPGN (unspecified), lipoprotein glomerulopathy (LipoN), autosomal dominant tubulointerstitial kidney disease (ADTKD), fibronectin nephropathy (Fibronectin N), and lupus nephritis (LN)

^d^Numbers in brackets showed the total number of disease-causing variants

### Distribution of *COL4As* variants

**Fig. 1:**
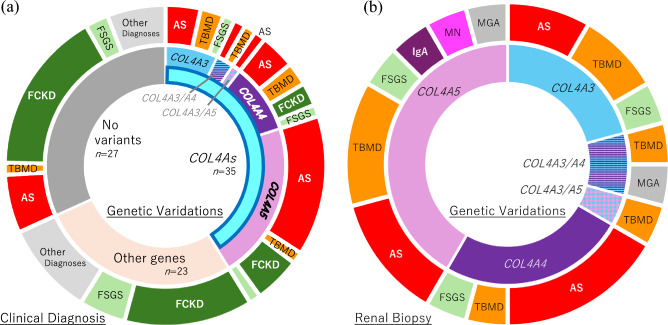
Relationship Between Genetic Mutations and Clinical and Pathological Diagnoses. **a** Distribution of pre-genetic clinical diagnoses among patients with mutations in *COL4As*, patients with other genetic mutations, and patients without significant genetic mutations. **b** Distribution of kidney biopsy results among patients with genetic mutations in *COL4As* who have undergone kidney biopsy. Among the patients diagnosed with AS due to *COL4A4* mutations, three cases were also identified with concurrent FSGS, IgA nephropathy, and medullary cystic kidney disease but were classified as Alport syndrome. For *COL4A5* mutations, one case was identified with concurrent Alport syndrome and FSGS, and another with thin basement membrane disease and FSGS, but they were classified as Alport syndrome and thin basement membrane disease, respectively

The results of genetic analysis among the 85 patients are shown in Fig. 1. Disease-causing variants were detected in 58 of the 85 (68.3%) patients. Of these, 35 patients (41.2%) had pathogenic *COL4As* variants (Table S3). Thirty-eight potentially pathogenic variants were identified in patients from the *COL4As* group including: 9 (25.7%) in *COL4A3*, 10 (28.6%) in *COL4A4*, and 19 (54.3%) in *COL4A5* (including duplications). Additionally, 23 patients (25.8%) had variants in genes other than *COL4As*. As detailed in Table S4, diagnostic variants in genes other than *COL4As* were found in 23 patients (27.1%). These included 3 cases of autosomal dominant tubulointerstitial kidney disease (*UMOD*), 3 cases of hereditary FSGS/steroid resistant nephrotic syndrome (*INF2* × 2, *WT1* × 1), 3 cases of nail-patella-like renal disease (*LMX1B*), and 2 cases of MYH9-related disease.

### Clinical data on patients with *COL4As* variants

Among the 35 patients with pathogenic *COL4As* variants, 20 (57.1%) patients were clinically pre-diagnosed with Alport syndrome, 6 (17.1%) with thin basement membrane disease, 6 (17.1%) with familial clustering of kidney disease, and 3 (8.6%) with FSGS (Fig. 1a). Additionally, 25 (71.4%) of the patients underwent renal biopsy. Histologically, 17 (48.6%) patients were diagnosed with Alport syndrome/thin basement membrane disease and 3 (8.6%) with FSGS (Fig. 1b).

The genes were distributed as 9 (25.7%) for *COL4A3*, 10 (28.6%) for *COL4A4*, and 19 (54.3%) for *COL4A5*, including duplications*.* Regarding the genetic diagnosis of Alport syndrome, 7 (PT479, PT687, PT788, PT1098, PT1226, PT1244, and PT1330; 20.0%) had hemizygous variants in COL4A5, 1 (PT1198; 2.9%) had homozygous *COL4A4* variant, and 2 (PT904 and PT982; 5.7%) had compound heterozygous variants in either *COL4A3* or *COL4A4*. Digenic variants involving *COL4A* genes were identified in 3 patients: PT904 and PT1054 had heterozygous variants in *COL4A3* and *COL4A4*, respectively, and PT1311 had a heterozygous *COL4A3* variant and a hemizygous *COL4A5* variant. Combinations of variants in *COL4As* and other genes (Table S4) were also observed, such as *COL4A5* and *SLC22A12* (PT1065), *COL4A4* and *UMOD* (PT1101), *COL4A5* and *MYH9* (PT1153), and *COL4A4* and *APOE* (PT1243)*.*

### Age distribution of first manifestations and genetic diagnosis

**Fig. 2:**
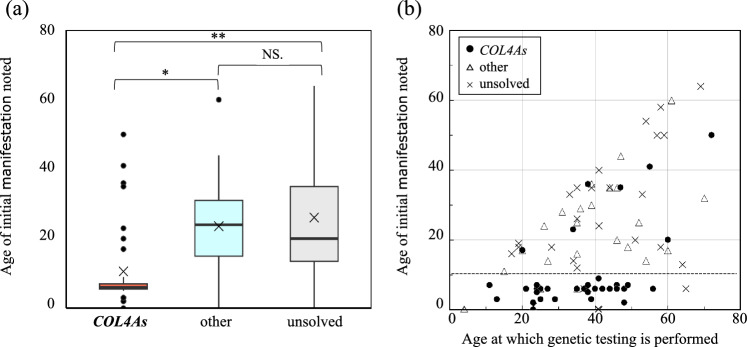
Age-related analysis of disease onset and genetic testing. **a** Age at disease onset for *COL4As*, other genes, and unsolved (no disease-causing variants) groups, depicted as a boxplot. Significant differences are indicated by *p = 0.0004 for *COL4As* vs. other genes, and **p = 0.0007 for *COL4As* vs. unsolved. NS denotes not significant differences (*p* = 0.76) between other genes and unsolved. **b** Relationship between age at which genetic test was performed and age at which first manifestations were noted. Black circle, *COL4As* cases; white triangle, non-*COL4A* disease-causing variant group; cross mark, unsolved (no disease-causing variants)

Figure 2a displays the age distribution when abnormal urinalysis was first noted among groups: *COL4As*, other variants (Table S4), and unsolved (no disease-causing variants). Notably, in the *COL4As* group, this manifestation was detected at the age of < 10 years in 27 (77.1%) out of 35 patients. The Kruskal–Wallis test indicated a statistically significant difference in median values among the groups (*H* = 19.87, *p* < 0.0001), with *COL4As* showing significantly lower median values compared to the other variants and unsolved groups. Specifically, the median age in *COL4As* was significantly lower than that in other variants (*U* = 164.5, *p* = 0.0004) and unsolved groups (*U* = 214.0, *p* = 0.0007), while no significant difference was observed between the other variants and unsolved groups (*U* = 294.5, *p* = 0.76). Furthermore, a substantial discrepancy was observed between age at first manifestation onset and age at genetic diagnosis, with means ± SD of 26.2 ± 12.8 years in *COL4As*, 15.4 ± 13.6 years in other genes, and 16.4 ± 17.2 years in the unsolved group (Fig. 2b).

### Association between *COL4As* variants and microscopic hematuria

The correlation between *COL4As* variants and the presence of microscopic hematuria is shown in Table 2. A significant association was found between *COL4As* variants positivity and the presence of microscopic hematuria before the age of 10 years (*χ*^2^ = 34.09, *p* < 0.001). Among the 35 patients with *COL4As* variants, 27 had detectable microscopic hematuria before the age of 10, indicating a sensitivity of 77.1%. Conversely, of the 50 patients without *COL4As* variants, including those without any pathogenic variants in other known genes, 44 tested negative for microscopic hematuria, demonstrating a specificity of 88.0%.

**Table 2:** The correlation between *COL4As* variants and the presence of microscopic hematuria within 85 patients with a family history of CKD

	*COL4As*(+)	*COL4As*(−)	Sum
MH(+) by 10 y.o	27	6	33
MH(−) by 10 y.o	8	44	52
Sum	35	50	85

MH, microscopic hematuria; *COL4As*( +) or *COL4As*(−), disease-causing variants in *COL4As* detected (+) or not (−), respectively

## Discussion

In this study, we examined patients with a family history of CKD, excluding cystic kidney disease which can be diagnosed from imaging. Despite reports of comprehensive genetic analysis focusing on kidney failure, CKD, younger patients, suspected Alport syndrome/thin basement membrane disease cases, and FSGS of histological diagnosis [18, 19], the significance of family history is particularly underscored. Groopman et al. [5] reported a substantial increase in diagnostic yield when patients had a known family history of kidney disease. They identified disease-causing variants in 9.3% of their patients. In our cohort, 68.2% (58 of 85) of the total patients had disease-causing variants. Domingo-Gallego et al. [6], who concentrated on early-onset CKD (< 30 years), identified disease-causing variants in 65% of the total, reinforcing the significance of considering additional clinical and genetic factors alongside family history. Thus, incorporating family history into the criteria for genetic screening in CKD can significantly enhance the identification of at-risk individuals, potentially leading to earlier and more precise interventions.

The school health checkup system in Japan, which is rare even globally [20], plays a critical role not only in early disease detection but also in screening for inherited kidney diseases that manifest urinary abnormalities from a young age. In our study, 77.1% of patients with *COL4As* variants had abnormal urinalysis including microscopic hematuria and proteinuria since childhood. Therefore, undergoing school health checkups, including urinalysis, by age 10 could substantially increase the likelihood of detecting genetic kidney diseases in children with a family history of CKD, as indicated by a positive likelihood ratio of 6.43. However, we did not genotype all individuals with abnormal urine findings from health examinations, yet the high prevalence of *COL4As* variants in adult CKD suggests that many individuals with microscopic hematuria detected during health screenings may carry these variants. Microscopic hematuria from an early age is common in diseases such as Alport syndrome and thin basement membrane disease, yet few large-scale studies have documented this; therefore, our study provides valuable data on this aspect.

Furthermore, as illustrated in Fig. 2b, there is a noticeable gap between the age when the urinary abnormalities were detected and the age when a genetic diagnosis was confirmed. This discrepancy highlights the potential delays in linking clinical findings with genetic insights, underscoring the need for more timely genetic testing in the diagnostic process. Historically, thin basement membrane disease, previously known as benign familial hematuria, lacked active follow-up due to the absence of significant kidney function decline, presenting mainly with blood in the urine [9, 21]. In 1996, Lemmink et al. linked autosomal recessive *COL4A3*/*COL4A4* AS genes with benign familial hematuria [22], a classification that has been reconsidered given recent findings of diminished kidney function in older patients initially diagnosed with thin basement membrane disease [23]. However, the term benign familial hematuria is no longer used in recent years [24]. Thus, the gap between clinical findings and genetic diagnosis can potentially miss critical treatment opportunities, underscoring the increasing importance of rapid genetic testing in clinical practice.

Pierides et al. (2009) documented significant progression to proteinuria and CKD in patients with similar genetic backgrounds. In our cohort, among 16 patients with *COL4A3* or *COL4A4* variants—including those with both genes affected—15 showed microscopic hematuria, all 16 developed proteinuria, and 13 had been noted to have microscopic hematuria during school urinalysis. This progression raises concerns about long-term kidney function deterioration [25]. Unfortunately, our study could not distinguish whether the first manifestations were hematuria or proteinuria. Typically, the initial findings in school health checkups, often just microscopic hematuria without proteinuria or kidney dysfunction, do not prompt immediate intervention. However, this results in missed follow-up opportunities, potentially delaying the detection of proteinuria and CKD progression.

This study underscores the need for further evaluation of the screening system's efficacy. Known potential treatment strategies for *COL4As*-related nephropathy include angiotensin-converting enzyme inhibitors, which may delay disease progression [26], the Nrf2 activator bardoxolone [27], and gene therapy for *COL4A5* mutations [28]. An early definitive genetic diagnosis can extend the time to kidney failure. As ours and other studies suggest, some kidney failure cases are likely due to *COL4As* variants, making therapeutic intervention crucial [28]. Nonetheless, the phenotypic expression of Alport syndrome is diverse [29], requiring careful consideration of whether to initiate treatment.

This study has several limitations. Firstly, patient diagnoses rely heavily on clinician judgment, which can sometimes be inconsistent. Secondly, the classification of disease-causing variants is based solely on previous reports, including ACMG criteria, MAF, and in silico pathogenicity prediction scores, which may not sufficiently capture the true pathological significance of these variants. Ideally, further analysis involving not only the proband but also blood relatives would help to validate co-segregation and strengthen the genetic evidence for candidate variants. However, this approach is often limited by the availability of only the proband’s DNA sample. Additionally, our genetic panel testing primarily targets exonic and splicing regions, the analysis of deep intronic regions and large genomic rearrangements is incomplete.

In conclusion, this study confirms the critical importance of family history in the identification of disease-causing variants, as is fundamental in genetic medicine. Except for cystic kidney disease, the prevalence of *COL4As*-related kidney diseases was high in families with a family history of CKD, and the majority of *COL4As* variant carriers had some abnormal test results, particularly microscopic hematuria, starting at a young age. The early identification of the condition, regular follow-up, and early therapeutic intervention at the right timing can help maintain a lifelong renal prognosis. Further studies in larger populations are needed as a starting point for precision medicine based on genetic data.

## Supplementary Information

Below is the link to the electronic supplementary material.

Supplementary file1 (DOCX 258 KB)

## Data Availability

The data that support the results of this study are available on request from the corresponding author. The data are not publicly available because of privacy or ethical restrictions.
