# Supplementary material for: Importance of family history and health checkup for school-aged children for type IV collagen-associated nephropathy in hereditary kidney disease
Source: J Nephrol. 2025 Aug 2;38(7):1909–16. doi: 10.1007/s40620-025-02355-w (PMC12484354; doi:10.1007/s40620-025-02355-w)
Supplement: Supplementary file 1 — Supplementary file1 (DOCX 258 KB) [file 40620_2025_2355_MOESM1_ESM.docx]

Item S1. Gene panel sequencing : Supplemental Method.

The 121 genes related to hereditary glomerular kidney diseases were selected and captured with customized biotinylated RNA capture probes using the SureDesign service (https://earray.chem.agilent.com/suredesign/home.htm). Pooled barcoded libraries were prepared from 40 ng of genomic DNA for NGS using SureSelect QXT kits (Agilent Technologies, Inc., CA, USA) according to the manufacturer’s instructions. Genomic DNA samples were prepared using the QIAamp DNA Blood Midi Kit (QIAGEN, KJ Venlo, Netherlands) according to the manufacturer’s instructions. The libraries were sequenced via SE 150-bp reads and an 8-bp barcode read using Illumina MiSeq sequencers. Original read files were aligned to a human reference sequence (UCSC hg19) using Burrows–Wheeler Aligner v.0.7.12,16 and SAM files were generated. The files were sorted and indexed using SAMtools (v.1.2).[S1, S2] The files were obtained in BAM format and were analyzed following the Genome Analysis Toolkit (GATK v3.7-0-gcfedb67) and best-practice guidelines.[S3] BAM files were subjected to (1) removal of duplicate reads (Picard MarkDuplicates v.1.119; https://github.com/broadinstitute/picard), (2) base-quality recalibration (GATK BaseRecalibrator v3.7-0-gcfedb67), and (3) single nucleotide variant detection, indel detection, and genotyping using GATK HaplotypeCaller (v3.7-0-gcfedb67). Copy-number variations (CNVs) were assessed using CONTRA.[S4]

Item S2. Variant filtering and classification : Supplemental method.

Variant annotation for the VCF files was completed using VEP (v82),[S5] GEMINI annotate (v0.18.0), and ANNOVAR table_annovar.pl program v.2016Feb1.[S6] For gene annotation, the RefSeq gene database (build hg19)[S7] was used. Variant annotation was based on the gnomAD dataset (https://gnomad.broadinstitute.org; v2.1.1) and the Integrative Japanese Genome Variation Database.[S8] Variants with minor allele frequencies (MAF) of >0.0005 and synonymous mutations were excluded. CADD,[S9] M-CAP,[S10] and GERP conservation scores[S11] were used to interpret the significance of the variants. HGMD Professional (version 2018.1; http://www.hgmd.cf.ac.uk/ac/) and ClinVar (https://www.ncbi.nlm.nih.gov/clinvar/) databases were used to evaluate known disease-causing variants.

Table S1. List of the genes screened in the study.

| 1 | *ABCG2* | 41 | *CLDN16* | 81 | *HNF1B* | 121 | *PROKR2* | 161 | *VDR* |
| --- | --- | --- | --- | --- | --- | --- | --- | --- | --- |
| 2 | *ACE* | 42 | *CLDN19* | 82 | *HNF4A* | 122 | *PTH* | 162 | *VHL* |
| 3 | *ACTN4* | 43 | *CNNM2* | 83 | *HOGA1* | 123 | *PTPRO* | 163 | *WNK1* |
| 4 | *ADAMTS13* | 44 | *COL4A3* | 84 | *HPRT1* | 124 | *REN* | 164 | *WNK2* |
| 5 | *AGTR1* | 45 | *COL4A4* | 85 | *HSD11B2* | 125 | *RET* | 165 | *WNK3* |
| 6 | *AGXT* | 46 | *COL4A5* | 86 | *INF2* | 126 | *SALL1* | 166 | *WNK4* |
| 7 | *ANLN* | 47 | *COL4A6* | 87 | *ITGA3* | 127 | *SARS2* | 167 | *WT1* |
| 8 | *ANOS1* | 48 | *COL5A1* | 88 | *ITGB4* | 128 | *SCNN1A* | 168 | *XDH* |
| 9 | *APOA5* | 49 | *COL5A2* | 89 | *KCNA1* | 129 | *SCNN1B* |  |  |
| 10 | *APOE* | 50 | *COQ2* | 90 | *KCNJ1* | 130 | *SCNN1G* |  |  |
| 11 | *APOL1* | 51 | *COQ6* | 91 | *KCNJ10* | 131 | *SGK1* |  |  |
| 12 | *APRT* | 52 | *COQ8B* | 92 | *KCNJ5* | 132 | *SIX1* |  |  |
| 13 | *AQP2* | 53 | *CRB2* | 93 | *KLHL2* | 133 | *SIX5* |  |  |
| 14 | *ARHGAP24* | 54 | *CTNS* | 94 | *KLHL3* | 134 | *SLC12A1* |  |  |
| 15 | *ARHGDIA* | 55 | *CUBN* | 95 | *LAMB2* | 135 | *SLC12A3* |  |  |
| 16 | *ASS1* | 56 | *CUL3* | 96 | *LMX1B* | 136 | *SLC16A12* |  |  |
| 17 | *ATP6V0A1* | 57 | *CYP11B1* | 97 | *LPL* | 137 | *SLC17A5* |  |  |
| 18 | *ATP6V0A4* | 58 | *CYP11B2* | 98 | *MAGED2* | 138 | *SLC22A12* |  |  |
| 19 | *ATP6V1B1* | 59 | *CYP27B1* | 99 | *MEFV* | 139 | *SLC2A2* |  |  |
| 20 | *ATP7B* | 60 | *DGKE* | 100 | *MEN1* | 140 | *SLC2A9* |  |  |
| 21 | *AVPR2* | 61 | *DMP1* | 101 | *Mitochondria* | 141 | *SLC34A1* |  |  |
| 22 | *BSND* | 62 | *EGF* | 102 | *MUC1* | 142 | *SLC34A3* |  |  |
| 23 | *C3* | 63 | *EHHADH* | 103 | *MYH9* | 143 | *SLC3A1* |  |  |
| 24 | *CA2* | 64 | *ENPP1* | 104 | *MYO1E* | 144 | *SLC4A1* |  |  |
| 25 | *CACNA1D* | 65 | *EYA1* | 105 | *NEDD4L* | 145 | *SLC4A4* |  |  |
| 26 | *CACNA1H* | 66 | *FBN1* | 106 | *NLRP3* | 146 | *SLC5A1* |  |  |
| 27 | *CASR* | 67 | *FGF23* | 107 | *NPHS1* | 147 | *SLC5A2* |  |  |
| 28 | *CD2AP* | 68 | *FGFR1* | 108 | *NPHS2* | 148 | *SLC7A9* |  |  |
| 29 | *CD46* | 69 | *FKBP9* | 109 | *NR3C2* | 149 | *SLC9A3* |  |  |
| 30 | *CDC73* | 70 | *FN1* | 110 | *OCRL* | 150 | *SMARCAL1* |  |  |
| 31 | *CDKN1B* | 71 | *FXYD2* | 111 | *OXSR1* | 151 | *SQSTM1* |  |  |
| 32 | *CFB* | 72 | *G6PC* | 112 | *PAX2* | 152 | *STK39* |  |  |
| 33 | *CFH* | 73 | *GATA3* | 113 | *PDSS2* | 153 | *STX16* |  |  |
| 34 | *CFHR1* | 74 | *GCM2* | 114 | *PHEX* | 154 | *THBD* |  |  |
| 35 | *CFHR3* | 75 | *GLA* | 115 | *PKD1* | 155 | *TNXB* |  |  |
| 36 | *CFHR5* | 76 | *GNA11* | 116 | *PKD2* | 156 | *TRPC6* |  |  |
| 37 | *CFI* | 77 | *GNAS* | 117 | *PKHD1* | 157 | *TRPM6* |  |  |
| 38 | *CLCN5* | 78 | *GNAS-AS1* | 118 | *PLCE1* | 158 | *TSC1* |  |  |
| 39 | *CLCNKA* | 79 | *GPIHBP1* | 119 | *PLG* | 159 | *TSC2* |  |  |
| 40 | *CLCNKB* | 80 | *GRHPR* | 120 | *PROK2* | 160 | *UMOD* |  |  |

Table S2. Family history in each patient

| 418 | Paternal uncles (HD) |
| --- | --- |
| 427 | Father (CKD), brother (CKD), sister (CKD) |
| 432 | Father (HD and hearing loss), paternal aunt (HD) |
| 456 | Mother (HD), 3 maternal aunts (HD) |
| 457 | Maternal uncle (HD), maternal grandfather (HD) |
| 465 | Father (HD) |
| 478 | Maternal grandmother (HD) |
| 479 | Mother (MH), sister (MH), maternal grandmother (HD) |
| 480 | Mother (CKD), maternal grandmother (CKD) |
| 507 | Child (MH) |
| 516 | Brother (MH), maternal grandfather (HD) |
| 539 | Brother (UP), Paternal grandfather (CKD) |
| 551 | Mother (HD) |
| 555 | Paternal grandfather (HD) |
| 557 | Son (MH), father (CKD), brother (MH), paternal grandmother (HD) |
| 558 | Daughter (UP), father (HD), sister (HD) |
| 565 | Mother (CKD), maternal aunt (CKD) |
| 568 | Paternal grandfather (CKD) |
| 669 | Brother (UP) |
| 687 | Mother (OB, UP), sister (OB, UP), maternal aunt (OB, UP) |
| 704 | Daughter (MH), father (OB, HD), sister (MH) |
| 706 | Mother (HD), maternal aunt (HD) |
| 724 | Father (HD), paternal grandfather (CKD) |
| 749 | Mother (CKD), maternal grandmother (OB, UP) |
| 776 | Daughter (MH), sister (MH) |
| 788 | Brother (MH), maternal grandfather (HD) |
| 795 | Paternal uncle (HD), paternal aunt (HD) |
| 797 | Father (HD), brother (CKD), paternal grandfather (HD) |
| 808 | Sister (UP) |
| 817 | Brother (HD) |
| 820 | Father (HD), brother (HD) |
| 844 | Paternal aunt (CKD) |
| 850 | Maternal aunt (UP) |
| 857 | Daughter (OB, UP), mother (HD), sister (OB, UP) |
| 886 | Sisters (CKD, HD) |
| 902 | Mother (MH), maternal grandfather (MH) |
| 904 | Mother (MH), brother (MH), paternal grandmother (MH) |
| 910 | Maternal uncle (MH) |
| 918 | Brother (HD) |
| 941 | Sister (CKD) |
| 943 | Mother (MH), brother (MH), maternal grandfather (HD) |
| 982 | Father (MH), paternal grandmother (MH) |
| 1025 | Son (HD), mother (HD), maternal uncle (HD) |
| 1030 | Son (HD), father (HD), sisters (MH), paternal grandfather (HD), paternal cousin (CKD) |
| 1045 | Mother (HD), maternal grandfather (HD) |
| 1054 | Mother (MH), maternal uncle (MH), maternal grandfather (HD) |
| 1065 | Father (HD) |
| 1069 | Father (CKD), paternal cousin (CKD), paternal uncle (HD) |
| 1081 | Father (HD), sister (UP), paternal relatives (HD) |
| 1084 | Mother (MH), sister (MH) |
| 1085 | Maternal uncle (HD), maternal aunt (HD) |
| 1098 | Son (daughter), maternal grandmother (HD) |
| 1101 | Brother (HD) |
| 1110 | Father (MH), sisters (MH) |
| 1120 | Son (MH), mother (MH), maternal uncle (HD) |
| 1133 | Mother (HD), maternal grandfather (CKD), maternal aunt (CKD) |
| 1140 | Father (HD) |
| 1153 | Maternal grandmother (HD) |
| 1154 | Mother (CKD), maternal uncle (HD), maternal aunt (CKD) |
| 1198 | Mother (MH), brother (HD), paternal grandmother (CKD) |
| 1203 | Mother (HD), brother (HD), maternal uncle (HD) |
| 1215 | Son (UP), Daughter (UP), father (HD), brother (UP), paternal uncle (HD) |
| 1218 | Mother (HD), maternal grandmother (HD) |
| 1221 | Mother (HD) |
| 1223 | Father (UP), brother (UP) |
| 1225 | Mother (CKD) |
| 1226 | Brother (HD), maternal relatives (CKD) |
| 1231 | Maternal grandmother (HD) |
| 1243 | Daughter (MH) |
| 1244 | Son (CKD), mother (CKD), brother (CKD) |
| 1251 | Sister (MH), paternal uncle (HD), paternal aunt (HD) |
| 1276 | Father (KT) |
| 1280 | Father (OB, CKD), mother (MH), paternal grandmother (HD) |
| 1282 | Mother (MH), maternal uncle (CKD), maternal grandmother (CKD) |
| 1305 | Father (HD), brother (MH), paternal uncle (HD) |
| 1311 | Mother (MH), maternal uncle (HD) |
| 1316 | Mother (HD) |
| 1318 | Mother (CKD), maternal uncle (HD), maternal uncle (CKD) |
| 1320 | Mother (HD), brother (HD) |
| 1328 | Father (HD), sister (CKD), paternal grandmother (CKD) |
| 1330 | Daughter (UP), mother (UP), sister (MH), maternal aunt (HD) |
| 1331 | Mother (HD), maternal grandfather (HD) |
| 1334 | Father (HD), brother (UP), paternal grandmother (CKD) |
| 1335 | Father (HD), maternal aunt (HD) |
| 1340 | Maternal grandmother (HD) |

CKD, chronic kidney disease; HD, hemodialysis; MH, microscopic hematuria; UP, urine protein.

Table S3. List of patients and disease-causing genetic variants in *COL4As* variants.

| Patient | Gene | Accession ID | DNA substitution | Amino acid substitution | Mutation type | CLINSIG | ClinVar2 | HGMD | 8.3KJPN | gnomAD | CADD | ACMG/AMP 2015 | Previous reports |
| --- | --- | --- | --- | --- | --- | --- | --- | --- | --- | --- | --- | --- | --- |
| PT 432 | *COL4A5* | NM_000495 | c.973G>A | p.325G>R | Heterozygous | Pathogenic | Pathogenic | DM | NA | NA | 28.9 | Likely pathogenic | [S44–S48] |
| PT 465 | *COL4A3* | NM_000091 | c.3464G>A | p.1155G>D | Heterozygous | NA | NA | DM | NA | NA | 26.7 | Uncertain significance | [S12], [S49], [S50] |
| PT 479 | *COL4A5* | NM_000495 | c.2965_2968delinsCTGAGTGCCCAGT | p.Asp989_Pro990delinsLeuSerAlaGlnSer | Hemizygous | NA | NA | NA | NA | NA | NA | NA | NA |
| PT 507 | *COL4A5* | NM_000495 | c.584G>A | p.195G>D | Heterozygous | Pathogenic | Pathogenic | DM | NA | NA | 26.0 | Uncertain significance | S51 |
| PT 557 | *COL4A3* | NM_000091 | c.2383G>T | p.795G>W | Heterozygous | NA | NA | NA | NA | NA | 30.0 | Uncertain significance | NA |
| PT 687 | *COL4A5* | NM_000495 | c.3427G>A | p.1143G>S | Hemizygous | Pathogenic | Pathogenic/ Likely_pathogenic | DM | NA | NA | 27.6 | Uncertain significance | [S52-S55] |
| PT 704 | *COL4A5* | NM_000495 | c.656G>A | p.219G>D | Heterozygous | NA | NA | NA | NA | NA | 26.8 | Uncertain significance | NA |
| PT 749 | *COL4A3* | NM_000091 | c.1909G>A | p.637G>R | Heterozygous | Uncertain significance | Conflicting interpretations of pathogenicity | NA | 0.0001 | 0.000008 | 26.1 | Uncertain significance | NA |
| PT 788 | *COL4A5* | NM_000495 | c.3586G>T | p.1196G>X | Hemizygous | NA | NA | DM | NA | NA | 45.0 | Pathogenic | [S53], [S56] |
| PT 817 | *COL4A4* | NM_000092 | c.327+1G>A |  | Heterozygous | NA | NA | NA | NA | NA | 33.0 | Pathogenic | NA |
| PT 902 | *COL4A5* | NM_000495 | c.1526G>A | p.509G>D | Heterozygous | NA | Pathogenic | DM | NA | NA | 26.1 | Uncertain significance | [S57], [S58] |
| PT 904 | *COL4A3* | NM_000091 | c.469G>C | p.157G>R | Heterozygous | NA | Uncertain significance | DM | NA | 0.000016 | 32.0 | Uncertain significance | [S59] |
| PT 904 | *COL4A3* | NM_000091 | c.4793T>G | p.1598L>R | Heterozygous | NA | Conflicting interpretations of pathogenicity | DM | 0.0004 | 0.000056 | 29.4 | Uncertain significance | [S49] |
| PT 941 | *COL4A5* | NM_000495 | c.2858G>T | p.953G>V | Heterozygous | Pathogenic | Conflicting interpretations of pathogenicity | DM? | 0.01 | 0.003602 | 26.9 | Likely benign | [S59-S70] |
| PT 943 | *COL4A4* | NM_000092 | c.2566C>T | p.856Q>X | Heterozygous | NA | NA | DM | NA | NA | 38.0 | Pathogenic | [S71] |
| PT 982 | *COL4A4* | NM_000092 | c.3307G>A | p.1103G>R | Heterozygous | NA | Likely pathogenic | DM | 0.0001 | 0.000020 | 25.5 | Uncertain significance | [S49] |
| PT 982 | *COL4A4* | NM_000092 | c.2165G>T | p.722G>V | Heterozygous | NA | NA | NA | NA | NA | 33.0 | Uncertain significance | NA |
| PT 1030 | *COL4A5* | NM_000495 | c.2999G>T | p.1000G>V | Heterozygous | Benign | Benign | DM? | NA | NA | 24.8 | Likely benign | [S65], [S72-S74] |
| PT 1054 | *COL4A3* | NM_000091 | c.3566G>A | p.1189G>E | Heterozygous | NA | NA | NA | NA | NA | 29.0 | Uncertain significance | NA |
| PT 1054 | *COL4A4* | NM_000092 | c.4817G>A | p.1606G>E | Heterozygous | NA | Uncertain significance | DM? | 0.0006 | 0.000036 | 27.2 | Uncertain significance | [S75] |
| PT 1065 | *COL4A5* | NM_000495 | c.3284G>A | p.1095G>D | Heterozygous | NA | NA | NA | NA | NA | 26.9 | Uncertain significance | NA |
| PT 1084 | *COL4A5* | NM_000495 | c.4804_4805del | p.H1602fs | Heterozygous | NA | NA | NA | NA | NA | 34.0 | Uncertain significance | NA |
| PT 1098 | *COL4A5* | NM_000495 | c.1781G>A | p.594G>D | Hemizygous | NA | Pathogenic | DM | NA | NA | 22.8 | Uncertain significance | [S57], [S76], [S77] |
| PT 1101 | *COL4A4* | NM_000092 | c.2045A>G | p.682D>G | Heterozygous | NA | Uncertain significance | DM | 0.0042 | 0.000072 | 22.2 | Uncertain significance | [S59] |
| PT 1110 | *COL4A3* | NM_000091 | c.943G>A | p.315G>S | Heterozygous | NA | NA | NA | 0.0001 | NA | 27.6 | Uncertain significance | NA |
| PT 1120 | *COL4A3* | NM_000091 | c.1918G>A | p.640G>R | Heterozygous | NA | Likely pathogenic | DM | NA | 0.000016 | 22.1 | Uncertain significance | [S78] |
| PT 1153 | *COL4A5* | NM_000495 | c.3940C>T | p.1314P>S | Heterozygous | NA | Conflicting interpretations of pathogenicity | DM | 0.0044 | 0.000323 | 23.7 | Likely benign | [S79-S81] |
| PT 1198 | *COL4A4* | NM_000092 | c.4847T>G | p.1616L>R | Homozygous | NA | NA | DM | 0.0002 | 0.000004 | 29.7 | Uncertain significance | [S82] |
| PT 1215 | *COL4A5* | NM_000495 | c.1075G>C | p.359G>R | Heterozygous | NA | NA | DM | NA | NA | 25.1 | Likely pathogenic | [S73] |
| PT 1226 | *COL4A5* | NM_000495 | c.2554G>A | p.852G>R | Hemizygous | Pathogenic | Pathogenic | DM | NA | NA | 31.0 | Uncertain significance | [S83] |
| PT 1243 | *COL4A4* | NM_000092 | c.2510G>C | p.837G>A | Heterozygous | NA | Likely pathogenic | DM | 0.0009 | 0.000008 | 25.0 | Uncertain significance | [S49], [S50], [S84] |
| PT 1244 | *COL4A5* | NM_000495 | c.5030G>A | p.1677R>Q | Hemizygous | Pathogenic | Pathogenic/Likely pathogenic | DM | NA | 0.000022 | 32.0 | Likely pathogenic | [S85-87] |
| PT 1276 | *COL4A3* | NM_000091 | c.2603G>T | p.868G>V | Heterozygous | NA | NA | NA | NA | NA | 24.3 | Uncertain significance | NA |
| PT 1282 | *COL4A5* | NM_000495 | c.1189G>A | p.397G>S | Heterozygous | NA | NA | NA | NA | NA | 27.1 | Uncertain significance | NA |
| PT 1305 | *COL4A4* | NM_000092 | c.1323_1340del | p.441_447del | Heterozygous | NA | NA | NA | NA | NA | 16.8 | Uncertain significance | [S88-S92] |
| PT 1311 | *COL4A3* | NM_000091 | c.1295C>T | p.432P>L | Heterozygous | NA | Uncertain significance | DM | 0.0018 | 0.000036 | 22.7 | Uncertain significance | [S59] |
| PT 1311 | *COL4A5* | NM_000495 | c.3410G>A | p.1137G>D | Hemizygous | NA | Pathogenic | DM | NA | NA | 26.9 | Uncertain significance | [S57] |
| PT 1330 | *COL4A5* | NM_000495 | c.3088G>A | p.1030G>S | Hemizygous | Pathogenic | Pathogenic | DM | NA | NA | 27.8 | Uncertain significance | [S56], [S74], [S86], [S93] |
| PT 1335 | *COL4A4* | NM_000092 | c.4847T>G | p.1616L>R | Heterozygous | NA | NA | DM | 0.0002 | 0.000004 | 29.7 | Uncertain significance | [S82] |

gnomAD, Genome Aggregation Database (v2.1.1, https://gnomad.broadinstitute.org); HGMD, Human Gene Mutation Database (https://www.hgmd.cf.ac.uk/ac/index.php); 8.3KJPN (Allele and genotype frequency panels from 8,380 Japanese individuals, released in August 31, 2020, https://jmorp.megabank.tohoku.ac.jp/202008/); ACMG, American College of Medical Genetics and Genomics; AMP, Association of Molecular Pathology; CADD, Combined Annotation Dependent Depletion; CLINSING, clinical significance; DM, damaging; NA, not available. Previous reports were listed in the Supplementary References.

Table S4. Variants list other than *COL4As*.

| Patient | Gene | Accession ID | DNA substitution | Amino acid substitution | Mutation type | CLINSIG | Clin Var2 | HGMD | 8.3KJPN | gnomAD | CADD | ACMG/AMP 2015 | Previous reports |
| --- | --- | --- | --- | --- | --- | --- | --- | --- | --- | --- | --- | --- | --- |
| 478 | NPHP4 | NM_015102 | c.2717G>A | p.906R>H | Heterozygous |  | - | DM | 0.0091 | 0.000160 | 9.4 | Uncertain significance | [S12–S15] |
| 478 | NPHP4 | NM_015102 | c.2198G>A | p.733G>D | Heterozygous | Uncertain significance | Uncertain_significance | DM | 0.0027 | 0.000075 | 24.3 | Uncertain significance | [S12–S16] |
| 539 | CLCN5 | NM_000084 | c.1561C>T | p.521L>F | Hemizygous | - | - | DM | - | - | 27.4 | Uncertain significance | [S17, S18] |
| 551 | FN1 | NM_002026 | c.4486G>A | p.1496V>M | Heterozygous |  |  |  | 0.0136 | 0.000099 | 27.7 | Uncertain significance |  |
| 555 | KLHL3 | NM_017415 | c.1660G>A | p.554V>M | Heterozygous | - | - | - | 0.0005 | 0.000004 | 27.6 | Likely pathogenic | - |
| 558 | TBHB | NM_000361 | c.1499C>T | p.T500M | Heterozygous |  |  | DM? | 0.0002 | 0.000017 | 15.9 | Uncertain significance |  |
| 565 | UMOD | NM_003361 | c.916T>A | p.306C>S | Heterozygous | - | - | DM | - | - | 23.7 | Uncertain significance | [S19] |
| 724 | UMOD | NM_003361 | c.121T>G | p.41C>G | Heterozygous | - | - | - | - | - | 24.1 | Uncertain significance | - |
| 795 | FBN1 | NM_000138 | c.226G>C | p.76G>R | Heterozygous | - | - | - | 0.0001 | - | 29.0 | Uncertain significance | - |
| 797 | UMOD | NM_003361 | c.404G>A | p.135C>Y | Heterozygous | - | - | - | - | - | 25.4 | Uncertain significance | - |
| 820 | TSC1 | NM_000368 | c.2485A>C | p.829S>R | Heterozygous | not provided, | Benign/Likely_benign | DM? | 0.0044 | 0.000080 | 27.5 | Uncertain significance | [S20] |
| 910 | WT1 | NM_024426 | c.1484G>A | p.495R>Q | Heterozygous | - | Uncertain_significance | - | - | - | 32.0 | Uncertain significance | - |
| 918 | APOE | NM_000041 | c.488G>C | p.163R>P | Heterozygous | Pathogenic | Pathogenic | DM | 0.0001(G>A) | - | 25.2 | Uncertain significance | [S21–S23] |
| 1065* | SLC22A12 | NM_144585 | c.269G>A | p.90R>H | Heterozygous | Pathogenic | Pathogenic | DM | 0.0038 | 0.000178 | 23.3 | Uncertain significance | [S24–S30] |
| 1081 | INF2 | NM_022489 | c.641G>A | p.214R>H | Heterozygous | Pathogenic | Pathogenic | DM | - | - | 25.1 | Uncertain significance | [S31–S33] |
| 1101* | UMOD | NM_003361 | c.193G>A | p.65D>N | Heterozygous | - | - | - | - | - | 25.5 | Uncertain significance | - |
| 1133 | WT1 | NM_024426 | c.1373G>A | p.458R>Q | Heterozygous | - | Uncertain_significance | DM | - | - | 32.0 | Uncertain significance | [S33–S35] |
| 1140 | MYH9 | NM_002473 | c.3215_3216insCG -  AGCT CCAGGC -  CCAGATCGC | p.A1072delins -  AELQAQIA | Heterozygous | Pathogenic | Pathogenic | - | - | - | 20.5 | Uncertain significance | - |
| 1153* | MYH9 | NM_002473 | c.5807G>A | p.1936R>Q | Heterozygous | - | - | - | - | 0.000012 | 22.4 | Uncertain significance | - |
| 1203 | LMX1B | NM_002316 | c.746G>A | p.249R>Q | Heterozygous | - | - | DM | - | - | 31.0 | Likely pathogenic | [S36, S37] |
| 1218 | INF2 | NM_022489 | c.530G>A | p.177R>H | Heterozygous | - | Pathogenic/Likely_pathogenic | DM | - | - | 26.5 | Uncertain significance | [S33, S38] |
| 1231 | APOE | NM_000041 | c.488G>C | p.163R>P | Heterozygous | Pathogenic | Pathogenic | DM | - | - | 25.2 | Uncertain significance | [S21–S23] |
| 1243* | APOE | NM_000041 | c.784G>A | p.262E>K | Heterozygous | Pathogenic | - | DM? | 0.0082 | 0.000205 | 23.3 | Uncertain significance | [S39] |
| 1243* | APOE | NM_000041 | c.787G>A | p.263E>K | Heterozygous | Pathogenic | - | DM? | 0.0082 | 0.000203 | 23.9 | Uncertain significance | [S39] |
| 1316 | TSC1 | NM_000368 | c.913+1G>A |  | Heterozygous | not provided | Pathogenic | DM | - | - | 36.0 | Pathogenic | [S40, S41] |
| 1318 | GLA | NM_000169 | c.829T>C | p.277W>R | Hemizygous | - | - | DM? | - | - | 22.6 | Uncertain significance | [S42] |
| 1320 | LMX1B | NM_002316 | c.655C>G | p.219P>A | Heterozygous | - | - | - | 0.0007 | 0.000004 | 25.6 | Uncertain significance | - |
| 1331 | MYH9 | NM_002473 | :c.1057G>A | p.353V>I | Heterozygous | - | - | DM? | - | 0.000068 | 20.7 | Uncertain significance | [S43] |
| 1334 | ARHGAP24 | NM_001025616 | c.825G>C | p.275Q>H | Heterozygous | - | - | - | 0.0001 | - | 23.8 | Uncertain significance | - |

gnomAD, Genome Aggregation Database; HGMD, Human Gene Mutation Database; 8.3KJPN, Allele and genotype frequency panels from 8,380 Japanese individuals, released in August 31, 2020; ACMG, American College of Medical Genetics and Genomics; AMP, Association of Molecular Pathology; CADD, Combined Annotation Dependent Depletion; CLINSING, clinical significance; DM, damaging; NA, not available. [12, 44–93]

Table S5. Clinical characteristics of the patients with *COL4As* variants.

|  | *COL4A3/A4/A5* | *COL4A3* | *COL4A4* | *COL4A5* |
| --- | --- | --- | --- | --- |
| Total | 35 | 9 | 10 | 19 |
| Homo | 1 | 0 | 1 | 0 |
| Comp het | 4 | 3 | 1 | 0 |
| Hemi | 8 | 0 | 0 | 8 |
| Female | 21 | 6 | 5 | 11 |
| Onset |  |  |  |  |
| Under 10 years | 28 | 8 | 7 | 16 |
| MH in urine | 33 | 8 | 10 | 18 |
| Urine protein | 32 | 9 | 10 | 16 |
| *Clinical Diagnosis* |  |  |  |  |
| AS | 20 | 5 | 4 | 13 |
| TBMD | 6 | 3 | 3 | 1 |
| FCKD | 6 | 0 | 2 | 4 |
| FSGS | 3 | 1 | 1 | 1 |
| *Kidney biopsy* |  |  |  |  |
| Total | 25 | 8 | 9 | 11 |
| AS/TBMD | 17 | 6 | 6 | 7 |
| FSGS | 6 | 1 | 2 | 3 |
| MGA | 1 | 0 | 0 | 1 |
| IgAN | 1 | 0 | 0 | 1 |

MH, microscopic hematuria; AS, Alport syndrome; Comp het, compound heterozygous; FCKD, familial clustering of kidney disease; FSGS, focal segmental glomerulonephritis; Hemi, hemizygous; Homo, homozygous; IgAN, IgA nephropathy; MGA, minor glomerular abnormalities; TBMD, thin basement membrane syndrome.

**Supplemental References**

S1. Li H, Handsaker B, Wysoker A, et al (2009) The Sequence Alignment/Map format and SAMtools. Bioinformatics 25:2078–2079. https://doi.org/10.1093/bioinformatics/btp352

S2. Li H, Durbin R (2009) Fast and accurate short read alignment with Burrows-Wheeler transform. Bioinformatics 25:1754–1760. https://doi.org/10.1093/bioinformatics/btp324

S3. McKenna A, Hanna M, Banks E, et al (2010) The Genome Analysis Toolkit: a MapReduce framework for analyzing next-generation DNA sequencing data. Genome Res 20:1297–1303. https://doi.org/10.1101/gr.107524.110

S4. Li J, Lupat R, Amarasinghe KC, et al (2012) CONTRA: copy number analysis for targeted resequencing. Bioinformatics 28:1307–1313. https://doi.org/10.1093/bioinformatics/bts146

S5. McLaren W, Gil L, Hunt SE, et al (2016) The Ensembl Variant Effect Predictor. Genome Biol 17:122. https://doi.org/10.1186/s13059-016-0974-4

S6. Wang K, Li M, Hakonarson H (2010) ANNOVAR: functional annotation of genetic variants from high-throughput sequencing data. Nucleic Acids Res 38:e164. https://doi.org/10.1093/nar/gkq603

S7. Pruitt KD, Brown GR, Hiatt SM, et al (2014) RefSeq: an update on mammalian reference sequences. Nucleic Acids Res 42:D756-63. https://doi.org/10.1093/nar/gkt1114

S8. Yamaguchi-Kabata Y, Nariai N, Kawai Y, et al (2015) iJGVD: an integrative Japanese genome variation database based on whole-genome sequencing. Hum Genome Var 2:15050. https://doi.org/10.1038/hgv.2015.50

S9. Kircher M, Witten DM, Jain P, et al (2014) A general framework for estimating the relative pathogenicity of human genetic variants. Nat Genet 46:310–315. https://doi.org/10.1038/ng.2892

S10. Jagadeesh KA, Wenger AM, Berger MJ, et al (2016) M-CAP eliminates a majority of variants of uncertain significance in clinical exomes at high sensitivity. Nat Genet 48:1581–1586. https://doi.org/10.1038/ng.3703

S11. Davydov EV, Goode DL, Sirota M, et al (2010) Identifying a high fraction of the human genome to be under selective constraint using GERP++. PLoS Comput Biol 6:e1001025. https://doi.org/10.1371/journal.pcbi.1001025

S12. Mori T, Hosomichi K, Chiga M, et al (2017) Comprehensive genetic testing approach for major inherited kidney diseases, using next-generation sequencing with a custom panel. Clin Exp Nephrol 21:63–75. https://doi.org/10.1007/s10157-016-1252-1

S13. Fujimaru T, Mori T, Sekine A, et al (2018) Kidney enlargement and multiple liver cyst formation implicate mutations in PKD1/2 in adult sporadic polycystic kidney disease. Clin Genet 94:125–131. https://doi.org/10.1111/cge.13249

S14. Takada D, Sekine A, Yabuuchi J, et al (2018) Renal histology and MRI in a 25-year-old Japanese man with nephronophthisis 4. Clin Nephrol 89:223–228. https://doi.org/10.5414/CN109175

S15. Sekine A, Fujimaru T, Hoshino J, et al (2019) Genotype-Clinical Correlations in Polycystic Kidney Disease with No Apparent Family History. Am J Nephrol 49:233–240. https://doi.org/10.1159/000497444

S16. Li S, Liu S, Chen W, et al (2018) A novel ZIC3 gene mutation identified in patients with heterotaxy and congenital heart disease. Sci Rep 8:12386. https://doi.org/10.1038/s41598-018-30204-3

S17. Cramer MT, Charlton JR, Fogo AB, et al (2014) Expanding the phenotype of proteinuria in Dent disease. A case series. Pediatr Nephrol 29:2051–2054. https://doi.org/10.1007/s00467-014-2824-5

S18. Solanki AK, Arif E, Morinelli T, et al (2018) A Novel CLCN5 Mutation Associated With Focal Segmental Glomerulosclerosis and Podocyte Injury. Kidney Int Rep 3:1443–1453. https://doi.org/10.1016/j.ekir.2018.06.003

S19. Kaminska-Pajak KA, Dyga K, Adamczyk P, et al (2016) Familial juvenile hyperuricemic nephropathy as rare cause of dialysis-dependent chronic kidney disease—a series of cases in two families. Ren Fail 38:1759–1762. https://doi.org/10.1080/0886022X.2016.1229991

S20. Kang KW, Kim W, Cho YW, et al (2019) Genetic characteristics of non-familial epilepsy. PeerJ 7:e8278. https://doi.org/10.7717/peerj.8278

S21. Oikawa S, Matsunaga A, Saito T, et al (1997) Apolipoprotein E Sendai (arginine 145-->proline): a new variant associated with lipoprotein glomerulopathy. J Am Soc Nephrol 8:820–823. https://doi.org/10.1681/ASN.V85820

S22. Georgiadou D, Stamatakis K, Efthimiadou EK, et al (2013) Thermodynamic and structural destabilization of apoE3 by hereditary mutations associated with the development of lipoprotein glomerulopathy [S]. J Lipid Res 54:164–176. https://doi.org/10.1194/jlr.M030965

S23. Toyota K, Hashimoto T, Ogino D, et al (2013) A founder haplotype of APOE-Sendai mutation associated with lipoprotein glomerulopathy. J Hum Genet 58:254–258. https://doi.org/10.1038/jhg.2013.8

S24. Iwai N, Mino Y, Hosoyamada M, et al (2004) A high prevalence of renal hypouricemia caused by inactive SLC22A12 in Japanese. Kidney Int 66:935–944. https://doi.org/10.1111/j.1523-1755.2004.00839.x

S25. Cheong HI, Kang JH, Lee JH, et al (2005) Mutational analysis of idiopathic renal hypouricemia in Korea. Pediatr Nephrol 20:886–890. https://doi.org/10.1007/s00467-005-1863-3

S26. Ishikawa I, Nakagawa M, Hayama S, et al (2005) Acute renal failure with severe loin pain and patchy renal ischaemia after anaerobic exercise (ALPE) (exercise-induced acute renal failure) in a father and child with URAT1 mutations beyond the W258X mutation. Nephrol Dial Transplant 20:1015. https://doi.org/10.1093/ndt/gfh751

S27. Inazu T, Kawahara T, Ishikawa I (2007) Rapid detection of R90H mutations in the human urate transporter 1 gene. Ann Clin Biochem 44:189–191. https://doi.org/10.1258/000456307780118028

S28. Ochi A, Takei T, Ichikawa A, et al (2012) A case of acute renal failure after exercise with renal hypouricemia demonstrated compound heterozygous mutations of uric acid transporter 1. Clin Exp Nephrol 16:316–319. https://doi.org/10.1007/s10157-011-0557-3

S29. Zhou Z, Ma L, Zhou J, et al (2018) Renal hypouricemia caused by novel compound heterozygous mutations in the SLC22A12 gene: a case report with literature review. BMC Med Genet 19:142. https://doi.org/10.1186/s12881-018-0595-8

S30. Zhou Z, Wang K, Zhou J, et al (2019) Amplicon targeted resequencing for SLC2A9 and SLC22A12 identified novel mutations in hypouricemia subjects. Mol Genet Genomic Med 7:e00722. https://doi.org/10.1002/mgg3.722

S31. Brown EJ, Schlöndorff JS, Becker DJ, et al (2010) Mutations in the formin gene INF2 cause focal segmental glomerulosclerosis. Nat Genet 42:72–76. https://doi.org/10.1038/ng.505

S32. Safarikova M, Stekrova J, Honsova E, et al (2018) Mutational screening of inverted formin 2 in adult-onset focal segmental glomerulosclerosis or minimal change patients from the Czech Republic. BMC Med Genet 19:147. https://doi.org/10.1186/s12881-018-0667-9

S33. Varner JD, Chryst-Stangl M, Esezobor CI, et al (2018) Genetic Testing for Steroid-Resistant-Nephrotic Syndrome in an Outbred Population. Front Pediatr 6:307. https://doi.org/10.3389/fped.2018.00307

S34. Hall G, Gbadegesin RA, Lavin P, et al (2015) A novel missense mutation of Wilms’ Tumor 1 causes autosomal dominant FSGS. J Am Soc Nephrol 26:831–843. https://doi.org/10.1681/ASN.2013101053

S35. Hall G, Gbadegesin RA, Lavin P, et al (2015) A Novel Missense Mutation of Wilms’ Tumor 1 Causes Autosomal Dominant FSGS. Journal of the American Society of Nephrology 26:831–843

S36. Hall G, Lane B, Chryst-Ladd M, et al (2017) Dysregulation of WTI (-KTS) is Associated with the Kidney-Specific Effects of the LMX1B R246Q Mutation. Sci Rep 7:39933. https://doi.org/10.1038/srep39933

S37. Edwards N, Rice SJ, Raman S, et al (2015) A novel LMX1B mutation in a family with end-stage renal disease of “unknown cause.” Clinical Kidney Journal 8:113–119

S38. Boyer O, Benoit G, Gribouval O, et al (2011) Mutations in INF2 are a major cause of autosomal dominant focal segmental glomerulosclerosis. J Am Soc Nephrol 22:239–245. https://doi.org/10.1681/ASN.2010050518

S39. Tada H, Kawashiri M-A, Nomura A, et al (2018) Oligogenic familial hypercholesterolemia, LDL cholesterol, and coronary artery disease. J Clin Lipidol 12:1436–1444. https://doi.org/10.1016/j.jacl.2018.08.006

S40. Yang G, Shi ZN, Meng Y, et al (2017) Phenotypic and genotypic characterization of Chinese children diagnosed with tuberous sclerosis complex. Clin Genet 91:764–768. https://doi.org/10.1111/cge.12920

S41. He J, Zhou W, Shi J, et al (2020) TSC1 and TSC2 Gene Mutations in Chinese Tuberous Sclerosis Complex Patients Clinically Characterized by Epilepsy. Genet Test Mol Biomarkers 24:1–5. https://doi.org/10.1089/gtmb.2019.0094

S42. Walsh R, Thomson KL, Ware JS, et al (2017) Reassessment of Mendelian gene pathogenicity using 7,855 cardiomyopathy cases and 60,706 reference samples. Genet Med 19:192–203. https://doi.org/10.1038/gim.2016.90

S43. Satterstrom FK, Kosmicki JA, Wang J, et al (2020) Large-Scale Exome Sequencing Study Implicates Both Developmental and Functional Changes in the Neurobiology of Autism. Cell 180:568-584.e23. https://doi.org/10.1016/j.cell.2019.12.036

S44. Gast C, Pengelly RJ, Lyon M, et al (2016) Collagen (COL4A) mutations are the most frequent mutations underlying adult focal segmental glomerulosclerosis. Nephrol Dial Transplant 31:961–970. https://doi.org/10.1093/ndt/gfv325

S45. Knebelmann B, Deschenes G, Gros F, et al (1992) Substitution of arginine for glycine 325 in the collagen alpha 5 (IV) chain associated with X-linked Alport syndrome: characterization of the mutation by direct sequencing of PCR-amplified lymphoblast cDNA fragments. Am J Hum Genet 51:135–142

S46. Wang F, Wang Y, Ding J, Yang J (2005) Detection of mutations in the COL4A5 gene by analyzing cDNA of skin fibroblasts. Kidney Int 67:1268–1274. https://doi.org/10.1111/j.1523-1755.2005.00204.x

S47. Srinivasan M, Uzel SGM, Gautieri A, et al (2009) Alport syndrome mutations in type IV tropocollagen alter molecular structure and nanomechanical properties. J Struct Biol 168:503–510. https://doi.org/10.1016/j.jsb.2009.08.015

S48. Gibson J, Gilbert RD, Bunyan DJ, et al (2013) Exome analysis resolves differential diagnosis of familial kidney disease and uncovers a potential confounding variant. Genet Res 95:165–173. https://doi.org/10.1017/S0016672313000220

S49. Oka M, Nozu K, Kaito H, et al (2014) Natural history of genetically proven autosomal recessive Alport syndrome. Pediatr Nephrol 29:1535–1544. https://doi.org/10.1007/s00467-014-2797-4

S50. Kamiyoshi N, Nozu K, Fu XJ, et al (2016) Genetic, clinical, and pathologic backgrounds of patients with autosomal dominant alport syndrome. Clin J Am Soc Nephrol 11:1441–1449. https://doi.org/10.2215/cjn.01000116

S51. Hertz JM, Juncker I, Persson U, et al (2001) Detection of mutations in the COL4A5 gene by SSCP in X-linked Alport syndrome. Hum Mutat 18:141–148. https://doi.org/10.1002/humu.1163

S52. Renieri A, Meroni M, Sessa A, et al (1994) Variability of clinical phenotype in a large Alport family with Gly 1143 Ser change of collagen alpha 5(IV)-chain. Nephron 67:444–449. https://doi.org/10.1159/000188020

S53. Xiong HY, Alipanahi B, Lee LJ, et al (2015) RNA splicing. The human splicing code reveals new insights into the genetic determinants of disease. Science 347:1254806. https://doi.org/10.1126/science.1254806

S54. Stapleton CP, Kennedy C, Fennelly NK, et al (2020) An Exome Sequencing Study of 10 Families with IgA Nephropathy. Nephron 144:72–83. https://doi.org/10.1159/000503564

S55. Savige J, Storey H, Il Cheong H, et al (2016) X-Linked and Autosomal Recessive Alport Syndrome: Pathogenic Variant Features and Further Genotype-Phenotype Correlations. PLoS One 11:e0161802. https://doi.org/10.1371/journal.pone.0161802

S56. Martin P, Heiskari N, Zhou J, et al (1998) High mutation detection rate in the COL4A5 collagen gene in suspected Alport syndrome using PCR and direct DNA sequencing. J Am Soc Nephrol 9:2291–2301. https://doi.org/10.1681/ASN.V9122291

S57. Hashimura Y, Nozu K, Kaito H, et al (2014) Milder clinical aspects of X-linked Alport syndrome in men positive for the collagen IV α5 chain. Kidney Int 85:1208–1213. https://doi.org/10.1038/ki.2013.479

S58. Fallerini C, Dosa L, Tita R, et al (2014) Unbiased next generation sequencing analysis confirms the existence of autosomal dominant Alport syndrome in a relevant fraction of cases. Clinical Genetics 86:252–257

S59. Miyagawa M, Naito T, Nishio S-Y, et al (2013) Targeted exon sequencing successfully discovers rare causative genes and clarifies the molecular epidemiology of Japanese deafness patients. PLoS One 8:e71381. https://doi.org/10.1371/journal.pone.0071381

S60. Knebelmann B, Breillat C, Forestier L, et al (1996) Spectrum of mutations in the COL4A5 collagen gene in X-linked Alport syndrome. Am J Hum Genet 59:1221–1232

S61. Berg JS, Adams M, Nassar N, et al (2013) An informatics approach to analyzing the incidentalome. Genet Med 15:36–44. https://doi.org/10.1038/gim.2012.112

S62. Lennon R, Stuart HM, Bierzynska A, et al (2015) Coinheritance of COL4A5 and MYO1E mutations accentuate the severity of kidney disease. Pediatr Nephrol 30:1459–1465. https://doi.org/10.1007/s00467-015-3067-9

S63. Miao Y, Xiong J, Zhang X, et al (2017) Genetic diagnosis of polycystic kidney disease, Alport syndrome, and thalassemia minor in a large Chinese family. Clin Sci 131:2427–2438. https://doi.org/10.1042/CS20170245

S64. Wang Y, Dang X, He Q, et al (2017) Mutation spectrum of genes associated with steroid-resistant nephrotic syndrome in Chinese children. Gene 625:15–20. https://doi.org/10.1016/j.gene.2017.04.050

S65. Azaiez H, Booth KT, Ephraim SS, et al (2018) Genomic Landscape and Mutational Signatures of Deafness-Associated Genes. Am J Hum Genet 103:484–497. https://doi.org/10.1016/j.ajhg.2018.08.006

S66. Ganesh S, Ahmed P H, Nadella RK, et al (2019) Exome sequencing in families with severe mental illness identifies novel and rare variants in genes implicated in Mendelian neuropsychiatric syndromes. Psychiatry Clin Neurosci 73:11–19. https://doi.org/10.1111/pcn.12788

S67. Zhang X, Zhang Y, Zhang Y, et al (2018) X-linked Alport syndrome: pathogenic variant features and further auditory genotype-phenotype correlations in males. Orphanet J Rare Dis 13:229. https://doi.org/10.1186/s13023-018-0974-4

S68. Landim-Vieira M, Johnston JR, Ji W, et al (2019) Familial Dilated Cardiomyopathy Associated With a Novel Combination of Compound Heterozygous TNNC1 Variants. Front Physiol 10:1612. https://doi.org/10.3389/fphys.2019.01612

S69. Le VS, Tran KT, Bui HTP, et al (2019) A Vietnamese human genetic variation database. Hum Mutat 40:1664–1675. https://doi.org/10.1002/humu.23835

S70. Sun Y, Xiang J, Liu Y, et al (2019) Increased diagnostic yield by reanalysis of data from a hearing loss gene panel. BMC Med Genomics 12:76. https://doi.org/10.1186/s12920-019-0531-6

S71. Akihisa T, Sato M, Wakayama Y, et al (2019) Glomerular Basement Membrane Protein Expression and the Diagnosis and Prognosis of Autosomal Dominant Alport Syndrome. Kidney Med 1:391–396. https://doi.org/10.1016/j.xkme.2019.06.007

S72. Kaneko K, Tanaka S, Hasui M, et al (2010) A family with X-linked benign familial hematuria. Pediatr Nephrol 25:545–548. https://doi.org/10.1007/s00467-009-1370-z

S73. Yamamura T, Nozu K, Fu XJ, et al (2017) Natural History and Genotype–Phenotype Correlation in Female X-Linked Alport Syndrome. Kidney International Reports 2:850–855. https://doi.org/10.1016/j.ekir.2017.04.011

S74. Kamura M, Yamamura T, Omachi K, et al (2020) Trimerization and Genotype-Phenotype Correlation of COL4A5 Mutants in Alport Syndrome. Kidney Int Rep 5:718–726. https://doi.org/10.1016/j.ekir.2020.01.008

S75. Baek J-I, Choi S-J, Park S-H, et al (2009) Identification of novel variants in the COL4A4 gene in Korean patients with thin basement membrane nephropathy. Indian J Med Res 129:525–533

S76. Schapiro D, Daga A, Lawson JA, et al (2019) Panel sequencing distinguishes monogenic forms of nephritis from nephrosis in children. Nephrol Dial Transplant 34:474–485. https://doi.org/10.1093/ndt/gfy050

S77. Yao T, Udwan K, John R, et al (2019) Integration of Genetic Testing and Pathology for the Diagnosis of Adults with FSGS. Clin J Am Soc Nephrol 14:213–223. https://doi.org/10.2215/CJN.08750718

S78. Heidet L, Arrondel C, Forestier L, et al (2001) Structure of the human type IV collagen gene COL4A3 and mutations in autosomal Alport syndrome. J Am Soc Nephrol 12:97–106. https://doi.org/10.1681/ASN.V12197

S79. Pan X, Yan J, Ren H, et al (2004) Detection of COL4A5 gene mutations in Chinese patients with Alport’s syndrome. Nephrol Dial Transplant 19:1944–1944. https://doi.org/10.1093/ndt/gfh360

S80. Liu J-H, Wei X-X, Li A, et al (2017) Novel mutations in COL4A3, COL4A4, and COL4A5 in Chinese patients with Alport Syndrome. PLoS One 12:e0177685. https://doi.org/10.1371/journal.pone.0177685

S81. Wang X, Li W, Wei K, et al (2018) Missense mutations in COL4A5 or COL4A6 genes may cause cerebrovascular fibromuscular dysplasia: Case report and literature review. Medicine 97:e11538. https://doi.org/10.1097/MD.0000000000011538

S82. Imafuku A, Nozu K, Sawa N, et al (2018) Autosomal dominant form of type IV collagen nephropathy exists among patients with hereditary nephritis difficult to diagnose clinicopathologically. Nephrology 23:940–947. https://doi.org/10.1111/nep.13115

S83. Kawai S, Nomura S, Harano T, et al (1996) The COL4A5 gene in Japanese Alport syndrome patients: Spectrum of mutations of all exons. Kidney Int 49:814–822. https://doi.org/10.1038/ki.1996.113

S84. Izumi Y, Hamaguchi A, Miura R, et al (2020) Autosomal dominant Alport syndrome due to a COL4A4 mutation with an additional ESPN variant detected by whole-exome analysis. CEN Case Rep 9:59–64. https://doi.org/10.1007/s13730-019-00429-w

S85. Barker DF, Denison JC, Atkin CL, Gregory MC (1997) Common ancestry of three Ashkenazi-American families with Alport syndrome and COL4A5 R1677Q. Hum Genet 99:681–684. https://doi.org/10.1007/s004390050429

S86. Pont-Kingdon G, Sumner K, Gedge F, et al (2009) Molecular testing for adult type Alport syndrome. BMC Nephrol 10:38. https://doi.org/10.1186/1471-2369-10-38

S87. Lata S, Marasa M, Li Y, et al (2018) Whole-Exome Sequencing in Adults With Chronic Kidney Disease: A Pilot Study. Ann Intern Med 168:100–109. https://doi.org/10.7326/M17-1319

S88. Domingo-Gallego A, Pybus M, Bullich G, et al (2022) Clinical utility of genetic testing in early-onset kidney disease: seven genes are the main players. Nephrol Dial Transplant 37:687–696. https://doi.org/10.1093/ndt/gfab019

S89. Zhu F, Li W, Li Z, et al (2018) Identification of a Novel COL4A4 Variant in Compound-Heterozygous State in a Patient With Alport Syndrome and Histological Findings Similar to Focal Segmental Glomerulosclerosis (FSGS). Front Genet 9:748. https://doi.org/10.3389/fgene.2018.00748

S90. Kamiyoshi N, Nozu K, Fu XJ, et al (2016) Genetic, Clinical, and Pathologic Backgrounds of Patients with Autosomal Dominant Alport Syndrome. Clin J Am Soc Nephrol 11:1441–1449. https://doi.org/10.2215/CJN.01000116

S91. Nabais Sá MJ, Storey H, Flinter F, et al (2015) Collagen type IV-related nephropathies in Portugal: pathogenic COL4A3 and COL4A4 mutations and clinical characterization of 25 families. Clin Genet 88:456–461. https://doi.org/10.1111/cge.12521

S92. Boye E, Mollet G, Forestier L, et al (1998) Determination of the genomic structure of the COL4A4 gene and of novel mutations causing autosomal recessive Alport syndrome. Am J Hum Genet 63:1329–1340. https://doi.org/10.1086/302106

S93. Warejko JK, Tan W, Daga A, et al (2018) Whole exome sequencing of patients with steroid-resistant nephrotic syndrome. Clin J Am Soc Nephrol 13:53–62. https://doi.org/10.2215/cjn.04120417
